# Cadmium Sulfide Quantum Dots Adversely Affect Gametogenesis in *Saccharomyces cerevisiae*

**DOI:** 10.3390/nano12132208

**Published:** 2022-06-27

**Authors:** Riccardo Rossi, Roberta Ruotolo, Giuseppe De Giorgio, Marta Marmiroli, Marco Villani, Andrea Zappettini, Nelson Marmiroli

**Affiliations:** 1 Department of Chemistry, Life Sciences and Environmental Sustainability, University of Parma, Parco Area delle Scienze 11/A, 43124 Parma, PR, Italy; riccardo.rossi1@unipr.it (R.R.); giuseppe.degiorgio@unipr.it (G.D.G.); marta.marmiroli@unipr.it (M.M.); 2 Institute of Materials for Electronics and Magnetism (IMEM), National Research Council (CNR), Parco Area delle Scienze 37/A, 43124 Parma, PR, Italy; marco.villani@imem.cnr.it (M.V.); andrea.zappettini@imem.cnr.it (A.Z.); 3 The Italian National Interuniversity Consortium for Environmental Sciences (CINSA), Parco Area delle Scienze 93/A, 43124 Parma, PR, Italy

**Keywords:** nanoparticles, quantum dots, nanomaterial toxicity, sporulation, gametogenesis, risk assessment

## Abstract

In the last decades, nanotechnology-based tools have attracted attention in the scientific community, due to their potential applications in different areas from medicine to engineering, but several toxicological effects mediated by these advanced materials have been shown on the environment and human health. At present, the effects of engineered nanomaterials on gametogenesis have not yet been well understood. In the present study, we addressed this issue using the yeast *Saccharomyces cerevisiae* as a model eukaryote to evaluate the effects of cadmium sulfide quantum dots (CdS QDs) on sporulation, a process equivalent to gametogenesis in higher organisms. We have observed that CdS QDs cause a strong inhibition of spore development with the formation of aberrant, multinucleated cells. In line with these observations, treatment with CdS QDs down-regulates genes encoding crucial regulators of sporulation process, in particular, the transcription factor Ndt80 that coordinates different genes involved in progression through the meiosis and spore morphogenesis. Down-regulation of *NDT80* mediated by CdS QDs causes a block of the meiotic cell cycle and a return to mitosis, leading to the formation of aberrant, multinucleated cells. These results indicate that CdS QDs inhibit gametogenesis in an irreversible manner, with adverse effects on cell-cycle progression.

## 1. Introduction

Nanotechnology represents a dynamic area of research and an economic field that is in significant expansion. Engineered nanomaterials (ENMs) have peculiar physico-chemical characteristics, which determine their mechanism of action and fate in the environment, making them different from their bulk materials [1]. Thus, the risk of exposure to ENMs keeps growing as their application expands [2,3]. Quantum dots (QDs) are semiconductor ENMs with peculiar optical and electrical properties [4,5], mainly due to their nanometer scale, with applications in several fields such as electronics and diagnostics [6]. In vitro and in vivo studies have shown that various types of QDs can induce cytotoxicity by different mechanisms [2,5,7,8,9,10], indicating that a safe assessment of these ENMs is essential to evaluate their potential applications. In yeast, cadmium selenide (CdSe) and cadmium sulfide (CdS) QDs impair mitochondrial function and cause oxidative stress [11,12,13], while cadmium telluride (CdTe) QDs cause toxicity inhibiting autophagy [14]. Ruotolo et al. (2018) reported that the toxicity of CdS QDs in yeast is strongly associated with the protein-corona formation on the nanoparticle (NP) surface, involving absorption of some proteins implicated in crucial metabolic pathways [15]. The interaction with the surface of CdS QDs induces a misfolded conformation of these “corona” proteins, which seems to mediate the adverse effects observed in yeast after treatment with these NPs.

At present, we still have limited information on the negative effects of ENMs on gametogenesis, a crucial metabolic process that leads to the formation of specialized haploid cells (gametes) through meiosis. Exposure to different types of QDs, carbon-based NPs, and metal-oxide NPs causes inflammation, oxidative stress, and DNA damage in mouse and human germ cells [16]. Talebi et al. (2013) reported that ZnO NPs reduce sperm number, cause sperm abnormality, and induce the formation of multinucleated giant cells [17]. Acute damage on mouse spermatogenesis in association with the formation of multinucleated giant cells was also observed upon treatment with curcumin-loaded NPs [18] and silver NPs [19]. Furthermore, it was demonstrated that exposure to cadmium oxide NPs results in renal injury in pregnant mice females and their newborn offspring [20].

In the present work, we have evaluated the effects of CdS QDs on gametogenesis, using *Saccharomyces cerevisiae* as the model organism. Although sporulation in yeast has specific peculiarities, several key steps of the gametogenesis process are conserved from yeast to higher organisms [21,22]. Upon nutrient starvation, diploid yeast cells undergo meiosis to generate four haploid spores, through a process tightly regulated by sequential transcriptional cascades, which is required for the temporal, coordinated expression of the three main groups of meiosis-specific genes (indicated as early, middle, and late genes) [23,24]. A starvation signal induces the expression of Ime1, the pivotal early regulator of the process, which in turn promotes the transcription of the genes required for entry into the S phase and progression through the meiotic prophase I. Among these genes, the transcription factor Ndt80 stimulates the middle genes required for exit from the pachytene and for progression through the two meiotic divisions [25,26,27]. Finally, the induction of meiosis-specific late genes leads to the synthesis of the gene products essential for spore formation and maturation [23].

In the present manuscript, we have analyzed the morphogenetic effects induced by exposure to CdS QDs on the sporulation process using fluorescent microscopy, flow cytometry, and environmental scanning electron microscopy (ESEM) analysis. To determine whether the morphological alterations caused by these ENMs were connected to an altered transcriptional reprogramming of the process, a set of meiosis-specific genes was analyzed by real-time PCR. Moreover, the potential role of the protein-corona formation on the surface of CdS QDs was investigated.

## 2. Materials and Methods

### 2.1. Preparation and Characterization of Nanoparticles

CdS QDs were synthesized by IMEM−CNR (Institute of Materials for Electronics and Magnetism; National Research Council, Parma, Italy), as previously described [28]. The mean diameter of the CdS QDs was 5 nm and their bulk density was 4.82 g cm^−3^. Zinc sulfide quantum dots (ZnS QDs; 5 nm, mean particle size) were also synthesized by IMEM-CNR (Parma, Italy), according to Zhao et al. (2004) [29]. Cerium oxide nanoparticles (CeO_2_ NPs) (<25 nm, particle size) and copper oxide nanoparticles (CuO NPs) (<50 nm, particle size) were purchased from Sigma-Aldrich (Merck, Darmstadt, Germany). Prior to use, NPs were resuspended in milliQ water and sonicated for 15 min at room temperature using a water-bath sonicator (Transsonic T460, Elma Schmidbauer GmbH, Singen, Germany) to reduce agglomeration of NPs. Average particle size (nm) of ENMs used in the present work was determined by transmission electron microscopy (TEM) analysis (Figure S1a), using a Talos F200S G2 SEM FEG (Thermo Fisher Scientific, Waltham, MA, USA). Hydrodynamic diameter (nm) and zeta potentials (mV) were determined by dynamic light scattering (DLS) analysis at room temperature (Figure S1b), using a Zetasizer Nano ZSP (Malvern Instruments, Malvern, UK). Additional information on QD synthesis and characterization are reported in the supplementary material (Supplementary Methods and Figure S2).

### 2.2. Yeast-Growth Conditions

All the experiments were performed using the diploid homotallic strain Z239-6B-6B (genotype: *α*/*a D lys2-1 ade2-1 ura3-3 his1 leu1-2 canR1*; the symbol *D* indicates the presence of the wild-type set of genes *HO*, *HMR* and *HML*) of *S. cerevisiae*. Vegetative cells were grown in YPD medium [1% (*w*/*v*) yeast extract, 2% (*w*/*v*) peptone, 2% (*w*/*v*) dextrose]. Spot assays (see below) were performed on selective SD (Synthetic Defined) agar plates [2% glucose (*w*/*v*), 6.7 g L^−1^ yeast nitrogen base, 30 mg L^−1^ lysine, 30 mg L^−1^ adenine, 50 mg L^−1^ uracil, 20 mg L^−1^ histidine, 30 mg L^−1^ leucine, 15 g L^−1^ agar]. For sporulation assay, yeast cells were precultured in YEPA medium (1% yeast extract, 2% peptone, 2% potassium acetate), washed in Phosphate-Buffered Saline (PBS), and subsequently resuspended in selective sporulation medium (2% potassium acetate, 30 mg L^−1^ lysine, 30 mg L^−1^ adenine, 50 mg L^−1^ uracil, 20 mg L^−1^ histidine, 30 mg L^−1^ leucine).

### 2.3. Cytotoxicity and Sporulation Assays

For spot assays, Z239-6B-6B strain was pre-grown for 24 h at 28 °C in YPD medium and then diluted to an optical density at 600 nm (OD_600_) of 1; ten-fold serial dilutions were made and 3 μL of each dilution were seeded in SD agar plates supplemented (or not) with CdS QDs (10–100 mg L^−1^). Plates were incubated at 28 °C and photographed after 48 h to evaluate cell growth.

To assess the toxicity of the NPs in liquid media, yeast cells were pre-grown for 24 h at 28 °C in YPD medium, then diluted to an OD_600_ of 0.1 in SD medium supplemented (or not) with CdS QDs (1–10 mg L^−1^), and incubated for 24 h at 28 °C.

For sporulation assays, yeast cells were grown at 28 °C for 24 h in YEPA medium, washed in PBS, and resuspended in sporulation medium (2 × 10^7^ cells mL^−1^, final concentration) with the supplementation of CdS QDs (1–10 mg L^−1^), ZnS QDs (1–100 mg L^−1^), CuO NPs (1–100 mg L^−1^), CeO_2_ NPs (1–20 mg L^−1^) or CdSO_4_ (0.005–1 mg L^−1^). Sporulation process was then monitored for 72 h at 28 °C.

### 2.4. Fluorescence Microscopy Analysis

To analyze the progression of sporulation, 1 × 10^7^ cells were collected by centrifugation, washed in water and fixed in 70% ethanol for 45 min. Cells were then stained with DAPI (4′,6-diamidino-2-phenylindole; excitation at 359 nm and emission at 457 nm) at a final concentration of 1 mg L^−1^ and washed in water two times prior to microscopy analysis. For the analysis of spore-wall formation, cells were collected by centrifugation, washed in water and stained with the chitin/chitosan-binding dye calcofluor white (CFW; excitation at 365 nm and emission at 435 nm) at a final concentration of 25 µM. To determine the percentage of dead cells after exposure to CdS QDs, yeast cells grown in sporulation medium were collected by centrifugation, washed in water, and stained with 100 mg L^−1^ of propidium iodide (PI; excitation at 536 nm and emission at 617 nm), a membrane-impermeant DNA dye excluded from viable cells, but internalized by dead cells. Zeiss Axio Imager.Z2 fluorescence microscope (Carl Zeiss Microscopy GmbH, Jena, Germany) was used for image acquisitions. For each condition, 150 cells in three independent experiments were analyzed.

### 2.5. Analysis of Spore Viability and Shape

To evaluate spore viability, a random-spore-analysis assay was performed by collecting spores from asci formed in the presence of low doses (1 mg L^−1^) of CdS QDs. Yeast cells were grown at 28 °C for 72 h in sporulation medium with the supplementation (or not) of CdS QDs; cells were then collected by centrifugation, washed in water, and resuspended in a solution containing 2-mercaptoethanol (143 mM, final concentration) and lyticase (2.4 KU/mL, final concentration). After incubation overnight at 28 °C with gentle shaking, Tween^®^ 80 was then added [0.1% (*v*/*v*), final concentration], and samples were subjected to several cycles of sonication (eight times for 1 min) using a water bath sonicator (Transsonic T460). Vigorous vortexing (four times) was then performed for 1 min (followed by 1 min incubation in ice) with a Thermo Savant FastPrep^®^ Cell Disrupter (Qbiogene Inc. Carlsbad, CA, USA). Lytic digestion was examined with optical microscopy analysis, and the procedure was repeated until the percentage of single spores reached 90%. The concentration of single spores in several samples was then determined with a hemocytometer, and 100 spores were seeded in YPD agar plates. Spore germination was evaluated after 72 h of incubation at 28 °C by colony formation on agar plates.

The shape and size of spores isolated from untreated or CdS QD-treated cells (1 mg L^−1^) were analyzed using environmental scanning-electron microscope (ESEM) Quanta^TM^ 250 FEG (FEI, Hillsboro, OR, USA), operating in wet mode. After isolation from asci, spores were washed and resuspended in water. The analysis was performed with an accelerating voltage of 15 kW, final lens aperture of 30 µm, and working distance (WD) of 7 mm. The humidity was set to 100% (at 3 °C).

### 2.6. Gene Expression Analysis

Yeast cells grown in sporulation medium with (or without) the supplementation of CdS QDs (4 mg L^−1^) were collected at different times (3, 8, and 24 h), and total RNA purification was performed using the RNeasy Mini Kit (Qiagen, Hilden, Germany), in accordance with the instructions of the manufacturer. Briefly, total RNA (1 µg) was reverse-transcribed with QuantiTect^®^ Reverse Transcription Kit (Qiagen), and cDNA was quantified by real-time PCR using the PowerUp SYBR™ Green Master Mix (Life Technologies, Carlsbad, CA, USA) and the ABI PRISM 7000 Sequence Detection System (Life Technologies). Relative quantification of gene expression was performed using the “comparative C_T_ method” and *ACT1* as housekeeping gene. The sequences of PCR primers used in this manuscript were indicated in Table 1.

### 2.7. Flow Cytometry Analysis

To prepare samples for flow-cytometry (FC) analysis, cells were grown in YEPA medium at 28 °C for 24 h, collected by centrifugation, washed in PBS, and resuspended in sporulation medium to a final concentration of 2 × 10^7^ cells mL^−1^ with (or without) the supplementation of CdS QDs (5 mg L^−1^). Cells were collected at different times (0, 6, 24, and 48 h), sonicated to break up cell clumps, fixed in ice-cold ethanol (70%) and conserved at −20 °C. Before analysis, samples were washed in water, resuspended in 50 mM Tris-HCl buffer (pH 7.5), and treated with RNase A (10 µg mL^−1^, final concentration) for 2 h at 37 °C. Cells were then resuspended in the FC buffer (180 mM NaCl, 100 mM Tris-HCl pH 8) and incubated with PI (2 µM, final concentration) for 30 min before analysis. Viable cells were identified by gating the unstained (PI^−^) population. As a positive control for the quantification of dead cells, aliquots of yeast cells (treated or not with CdS QDs) were put at 95 °C for 10 min prior to PI staining. For the quantification of dead (PI^+^) cells, 20,000 cells from each sample were selected and analyzed using a NovoCyte^®^ flow cytometer (ACEA Biosciences Inc., San Diego, CA, USA).

### 2.8. In Vitro Protein-QD Binding Assay

Yeast cells were grown in YEPA medium at 28 °C for 24 h, washed in PBS, and resuspended in sporulation medium (2 × 10^7^ cells mL^−1^, final concentration). After 72 h at 28 °C without CdS QD treatment, yeast cells were collected by centrifugation, and total cell extracts were prepared as previously described [15]. The protein concentration of cell extracts was measured using the bicinchoninic acid assay (Pierce^TM^ BCA Protein Assay Kit; Thermo Fisher Scientific), in accordance with the instructions of the manufacturer.

To identify hard corona proteins adsorbed on the CdS QD surface, the cell lysate (7 g L^−1^, final concentration) obtained from sporulated cells (see before) was incubated for 24 h at 4 °C with gentle agitation in binding buffer [10% (*v*/*v*) glycerol, 100 mM NaCl, 5 mM MgCl_2_, 50 mM Tris HCl pH 7.4] with the addition of CdS QDs (0.5 g L^−1^, final concentration). Hard corona proteins were isolated and analyzed by liquid-chromatography-mass spectrometry (LC-MS/MS) using a Dionex Ultimate 3000 micro HPLC device, coupled to an LTQ-Orbitrap XL mass spectrometer (Thermo Fisher Scientific), according to Ruotolo et al. (2018).

### 2.9. Statistical Analysis

For each experiment, at least three biological replicates were performed. GraphPad Prism v6.0 software was used to perform the statistical analysis. One-way ANOVA, followed by Dunnett’s multiple comparisons test (* *p* < 0.05; ** *p* < 0.01; *** *p* < 0.001; **** *p* < 0.0001), was used to determine significant differences between samples. UniProt (www.uniprot.org) and SGD (https://www.yeastgenome.org/) databases were used for the retrieval of gene/protein information regarding hard corona proteins. NovoExpress software (Agilent Technologies, Santa Clara, CA, USA) was used to analyze cytofluorimetric data.

## 3. Results

### 3.1. Toxicity of CdS QDs in Different Growth Conditions

**Figure 1:**
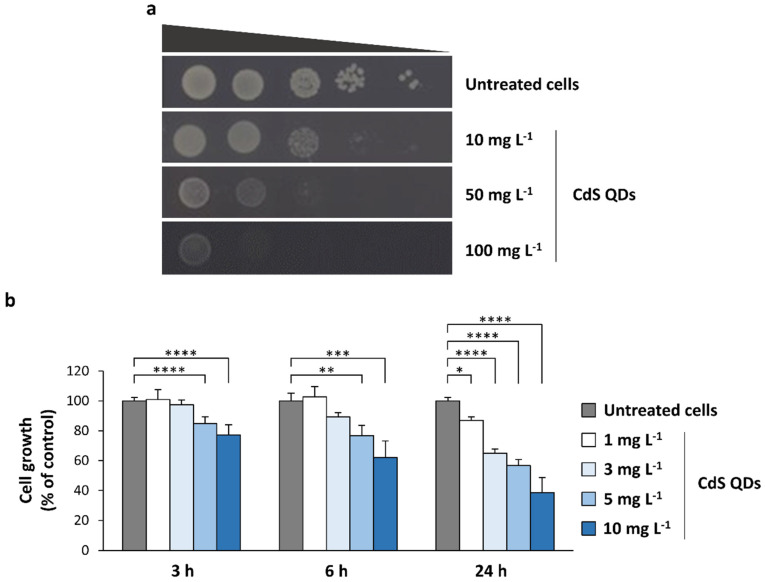
CdS QDs negatively affect the growth of Z239-6B-6B diploid strain in fermentative conditions. (**a**) Spot assay. Ten-fold serial dilutions (starting from OD_600_ = 1) of Z239-6B-6B diploid strain were seeded on SD agar plates supplemented with CdS QDs (10–100 mg L^−1^). Control (untreated) cells were also spotted on SD agar plates (without ENMs). Plates were incubated at 28 °C for 48 h. (**b**) Growth curves were recorded for 24 h in SD medium with (or without) the supplementation of CdS QDs (1–10 mg L^−1^). The values shown in the histograms are the mean (±standard deviation) of three independent experiments performed in triplicate. Significance was determined by one-way ANOVA with Dunnett’s multiple-comparisons test. * *p* < 0.05; ** *p* < 0.01; *** *p* < 0.001; **** *p* < 0.0001.

Different viability assays were performed to evaluate the fitness of the diploid yeast strain Z239-6B-6B after CdS QD treatment in fermentative conditions (SD medium). For spot assays, aliquots of 10-fold serial dilutions of yeast pre-cultures were seeded in SD agar plates supplemented (or not) with CdS QDs (10–100 mg L^−1^). We observed that concentrations of CdS QDs greater than 10 mg L^−1^ strongly reduced the viability of the diploid strain (Figure 1a), similarly to what was observed for haploid yeast cells [15]. To better define the range of CdS QD concentrations (≤10 mg L^−1^) that cause a growth impairment in diploid cells, growth curves were recorded for 24 h in SD medium with (or without) the supplementation of these NPs (Figure 1b). In SD medium, the exposure to the higher concentration tested of CdS QDs (10 mg L^−1^) causes a decrease in the yeast viability, greater than 50% as compared to the control (untreated) cells (Figure 1b).

**Figure 2:**
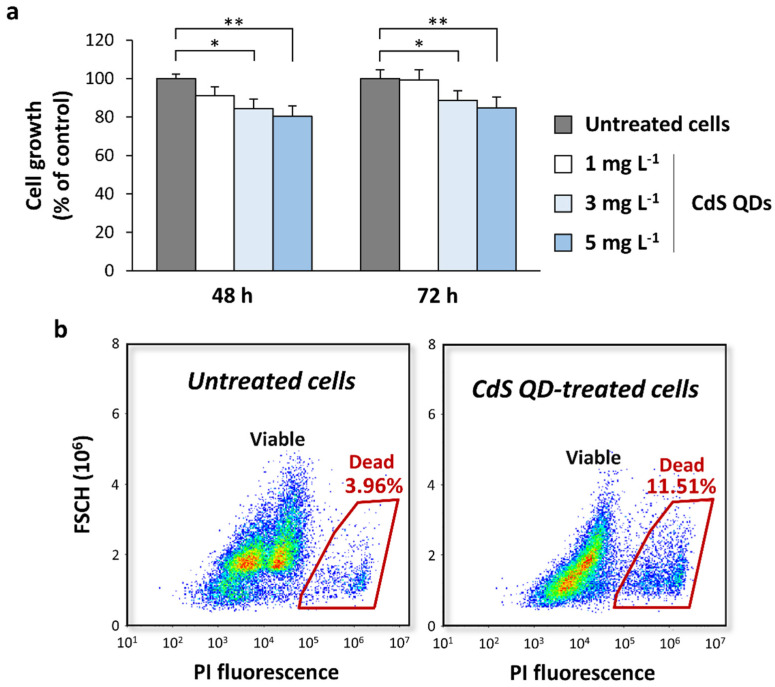
CdS QDs do not significantly alter the yeast growth in sporulation medium. (**a**) Z239-6B-6B diploid strain was grown in sporulation medium supplemented with CdS QDs (1–10 mg L^−1^), and growth curves were recorded for 48 h and 72 h at 28 °C. The results are expressed as percentage of cell growth (OD_600_) of CdS QD-treated cells relative to the control (untreated) cells (set to 100%). The values shown in the histograms are the mean (±standard deviation) of three independent experiments performed in triplicate. Significance was determined by one-way ANOVA with Dunnett’s multiple-comparisons test. * *p* < 0.05; ** *p* < 0.01. (**b**) Cell viability was also determined using flow-cytometry (FC) analysis. After CdS QD treatment (5 mg L^−1^), the cells were stained with propidium iodide (PI) and analyzed with FC. Representative dot-plots of forward-scatter signals (FSCH) versus PI fluorescence intensity are shown, and the percentages of dead (PI^+^) cells were indicated for each sample. Significance was determined by *t*-test (*p* < 0.05).

Then, we evaluated the inhibitory effect of CdS QDs (at doses of 1–5 mg L^−1^) on the yeast growth in a medium that induces the sporulation in diploid cells after 72 h. The exposure to CdS QDs caused a lower inhibition of the yeast growth in the sporulation medium (Figure 2a), compared to the fermentative conditions (Figure 1b). In fact, it is known that the sporulation process produces spores that are more resistant to environmental stresses than vegetative cells. To further evaluate if the treatment with CdS QDs did not significantly affect the viability of the cells under sporulation conditions, an experiment was conducted staining the yeast cells with PI (Figure 2b), a dye that allows the discrimination of viable (PI^−^) and dead (PI^+^) cells. The percentage of viable cells in the sporulation medium obtained by cytofluorimetric analysis in the control (untreated) cells was about 96% of the total cells (Figure 2b). The treatment with CdS QDs at the highest doses tested (5 mg L^−1^) slightly reduced the percentage of viable cells after 72 h (by about 8%; Figure 2b), in line with what was observed in the growth curves (Figure 2a).

### 3.2. CdS QDs Strongly Affect the Sporulation Process

**Figure 3:**
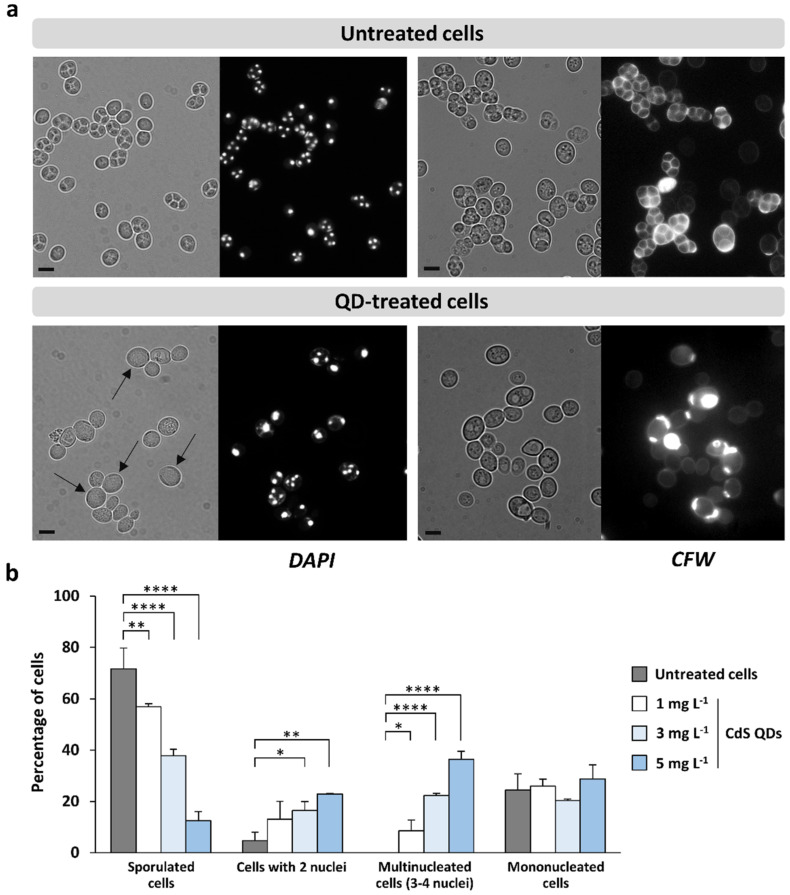
CdS QDs strongly affect the sporulation process. (**a**) Z239-6B-6B diploid strain was grown in sporulation conditions in the presence (or not) of CdS QD treatment (1–5 mg L^−1^); after 72 h, cells were then stained with DAPI and calcofluor white (CFW) and analyzed using fluorescence microscopy. CdS QD treatment caused the formation of asci containing three or four nuclei but with the absence of refractile spores (multinucleated cells, indicated by black arrows). Representative images (scale bars, 5 µm) of untreated and CdS QD-treated (5 mg L^−1^) cells are shown. (**b**) The percentage of cells (relative to the total number of cells stained with DAPI) representative of each phenotypic category was quantified. The values shown in the histograms are the mean (±standard deviation) of three independent experiments performed in triplicate. Significance was determined by one-way ANOVA with Dunnett’s multiple comparisons test. * *p* < 0.05; ** *p* < 0.01; **** *p* < 0.0001.

To evaluate the effects of CdS QDs in meiotic-nuclear division and spore morphogenesis, yeast cells were grown in sporulation medium with the supplementation of CdS QDs (1–5 mg L^−1^) and analyzed with optical microscopy. After 72 h of treatment, cells were stained with DAPI and calcofluor white to monitor, respectively, the nuclear divisions and the spore-wall formation by fluorescence microscopy (Figure 3a). Microscope observations revealed four different categories of phenotypes (Figure 3b): (i) cells which had correctly completed gametogenesis and presented asci with four refractile spores (each containing the corresponding haploid nucleus), representing ~70% of untreated cells; (ii) cells containing two nuclei (and no spore formation), a phenotype that significantly increased after treatment with higher doses of CdS QDs; (iii) cells with three (or four) distinct nuclei but without spore formation (multinucleated cells), a phenotype observed only in cells treated with CdS QDs; and (iv) mononucleated cells that did not undergo meiosis.

This analysis showed that exposure to the highest concentrations (3 and 5 mg L^−1^) of CdS QDs caused a strong alteration of the meiotic-nuclear divisions, in combination with the inhibition of spore morphogenesis, resulting in the formation of aberrant asci containing the meiotic products, but without the formation of refractile spores (in particular, multinucleated cells with three or four nuclei; Figure 3). This phenotype was also observed to a lesser extent (~10% of treated cells; Figure 3b) at the lowest dose of CdS QDs but not in untreated cells, underlining that the formation of multinucleated cells can highlight a molecular alteration of the gametogenesis induced by CdS QD treatment. In the literature, a similar phenotype was observed in yeast strains mutated in some meiosis-specific genes, which encode the proteins required for prospore-membrane morphogenesis (e.g., *spo1Δ* and *spo71Δ*) [30,31]. Moreover, it was also demonstrated that a reduced expression of *NDT80*, a gene encoding a key regulator of middle–late meiotic genes, and *SMK1*, encoding a sporulation-specific mitogen-activated protein kinase (MAPK), regulated by Ndt80, leads to the formation of multinucleated cells [24,32,33].

**Figure 4:**
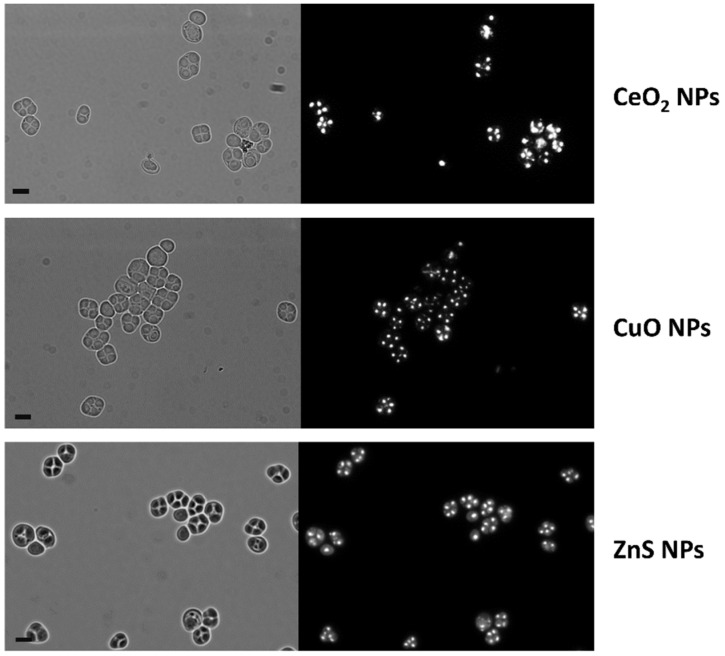
Effect of metal-based NPs on sporulation process. Yeast cells were grown in sporulation medium at 28 °C for 72 h in the presence of high doses of CeO_2_ NPs (20 mg L^−1^), CuO NPs (100 mg L^−1^), and ZnS QDs (100 mg L^−1^). Yeast cells were then stained with DAPI and analyzed using fluorescence microscopy. Representative images are shown. Scale bars, 5 µm.

To elucidate if the morphological and nuclear alterations of the gametogenesis of *S. cerevisiae* induced by CdS QDs were specific for these ENMs, the same sporulation assay was also carried out using other metal-based nanoparticles characterized by different chemical compositions, sizes, and zeta potentials (Figure S1): ZnS QDs, to evaluate QDs with a different metal composition; CuO NPs, to evaluate ENMs with potential applications from biomedicine to agriculture [34,35,36], but for which several studies have reported toxicity in different organisms [37,38,39]; and CeO_2_ NPs, to evaluate ENMs with very low toxicity, which in yeast have been shown to inhibit toxicity induced by α-synuclein in a yeast model of Parkinson’s disease [40]. We have observed that even at much higher doses than those used for CdS QDs, none of these ENMs significantly affected the sporulation process in *S. cerevisiae* (Figure 4).

**Figure 5:**
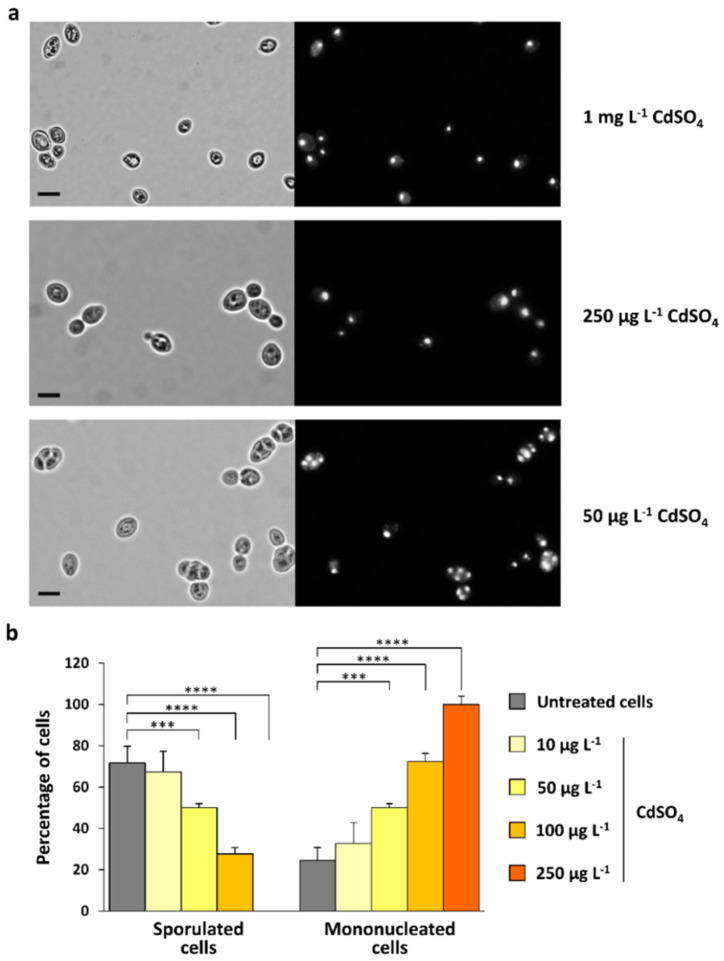
Effect of cadmium ions on sporulation process. (**a**) Yeast cells were grown at 28 °C for 72 h in sporulation medium in the presence of CdSO_4_ (0.01–1 mg L^−1^); cells were then stained with DAPI and analyzed using fluorescence microscopy. Representative images are shown. Scale bars, 5 µm. (**b**) The percentage of cells (relative to the total number of cells stained with DAPI) representative of each phenotypic category was quantified. The values shown in the histograms are the mean (±standard deviation) of three independent experiments performed in triplicate. Significance was determined by one-way ANOVA with Dunnett’s multiple-comparisons test. *** *p* < 0.001; **** *p* < 0.0001.

We have also evaluated the effect of cadmium (CdSO_4_) treatment on sporulation, using doses of metal ions equivalent to those used for the treatments with CdS QDs. We have observed that Cd(II) exposure strongly affected the sporulation process, but in a different manner than the CdS QDs. Unlike with CdS QDs, the sporulation process is completely inhibited by Cd(II) exposure even at doses equivalent to the lowest concentration of CdS QDs tested (1 mg L^−1^). In these conditions, no formation of multinucleated cells was observed (Figure 5a).

As we have previously observed, when low amounts of Cd(II) ions were released from CdS QDs into culture media [15,41,42], we have evaluated the potential inhibitory effect of Cd(II) on the sporulation process even at much lower doses than those used for CdS QDs (Figure 5). We have observed that sporulation was not inhibited at the metal concentration (5 µg L^−1^) corresponding to the estimated dissolution percentage (0.1%) of Cd(II) from CdS QDs [15]. At higher doses of Cd(II), ranging from 10–100 µg L^−1^, yeast sporulation was partially inhibited by Cd(II) treatment, with a progressive increase in percentage of mononucleated cells (which do not undergo meiosis) compared to the control cells. At concentrations greater than 250 µg L^−1^, Cd(II) treatment completely inhibited the sporulation process and only mononucleated cells were observed.

It is important to note that, unlike with Cd(II), CdS QD treatments did not show an increase in the percentage of mononucleated cells compared to the control cells, but only in the formation of aberrant, multinucleated cells (Figure 3b). These results suggest that the morphological abnormalities observed in yeast sporulation after exposure to CdS QDs cannot be simply explained by the presence of dissolved metal from the NPs, but they appear to be associated with an effect of these materials in their nanoform.

### 3.3. CdS QDs Adversely Affect Spore Germination and Morphology

A random spore analysis was performed to evaluate the integrity and viability of the single spores formed as a consequence of the meiotic process in the presence of low doses (1 mg L^−1^) of CdS QDs, taking into account the fact that most of the cells (~60%) treated in this condition consisted of asci (containing four spores), which seem indistinguishable from those found in untreated cells (Figure 3). It was not possible to perform this analysis with cells exposed to higher concentrations of CdS QDs because the spore morphogenesis is strongly compromised by the treatment at these doses (Figure 3b). Untreated asci and asci treated with CdS QDs (1 mg L^−1^) were collected after 72 h of growth in the sporulation medium. Given that not all cells undergo meiosis, samples may contain vegetative cells (mononucleated cells) in addition to asci (Figure 3b). To eliminate these vegetative cells, and at the same time to release spores from asci, the sporulation mixture was treated with lyticase, an enzyme that lyses the cell walls of asci and vegetative cells, without affecting the viability of spores. Spores were recovered and seeded in plates containing glucose-containing rich medium (YPD), to evaluate their competence to germinate and form colonies after three days of incubation at 28 °C. We have observed that the rate of spore germination was two-fold lower in the CdS QD-treated cells (30%) compared to the untreated sample (61%). These results indicate that, though most of the yeast cells (~60%; Figure 3b) completed gametogenesis with no evident morphological differences observed by fluorescence microscopy (with the exception of 10% of cells with a “multinucleated” phenotype), the viability of single spores appears to be affected by CdS QDs at the lowest doses tested.

**Figure 6:**
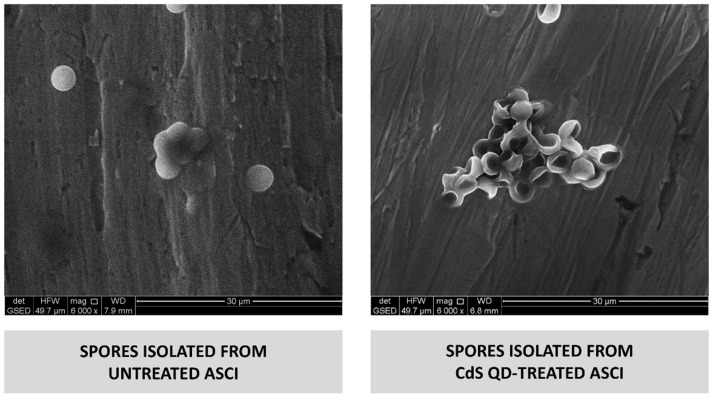
Morphology of spores isolated from untreated and CdS QD-treated (1 mg L^−1^) asci. Samples were maintained in water prior to observation on machined commercially pure titanium at 6000× magnification, using environmental scanning-electron microscopy (ESEM) in wet mode.

Spores isolated from untreated and CdS QD-treated asci (at the concentration of 1 mg L^−1^) were also analyzed, using ESEM in wet mode to evaluate their morphology and size (Figure 6). We have observed that although the spore size was nearly identical in all samples analyzed, the shape of the spores isolated from the asci treated with the lowest doses of CdS QDs tested was strongly altered (Figure 6). Spores isolated from untreated asci have the expected rounded shape, while the spores isolated from the CdS QDtreated samples are all “crumpled”. Defects observed in the germination rate of the spores isolated from the asci treated with the lowest dose of CdS QDs may result from these dramatic changes in the spore morphology.

### 3.4. CdS QDs Cause the Transcriptional Repression of Middle–Late Genes Involved in the Sporulation Process

**Figure 7:**
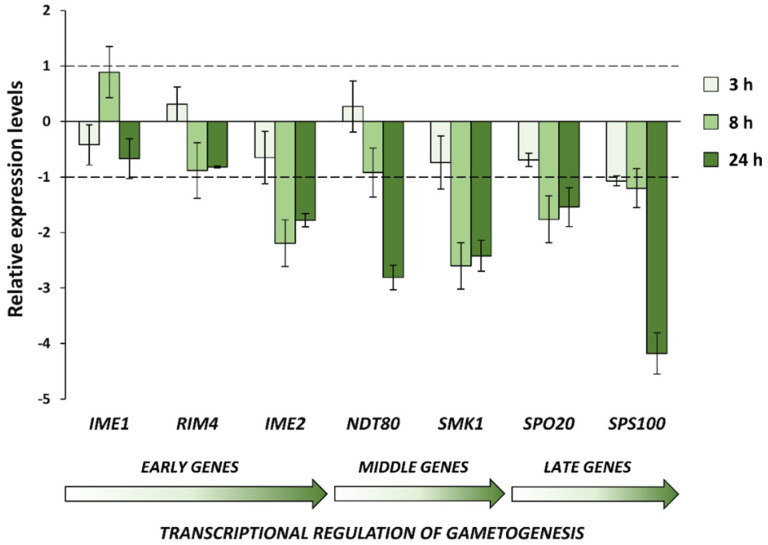
CdS QDs affect the expression of sporulation-specific genes. Cells were grown in sporulation medium for different time periods (3, 8, and 24 h) in the presence of CdS QDs (4 mg L^−1^). Control (untreated) cells grown in the same conditions were used as reference sample. Total RNA was extracted from each sample, equivalent amounts were reverse-transcribed and analyzed by real-time PCR. Transcript levels were normalized to the housekeeping gene *ACT1*. Data represent the relative expression levels (Log_2_-transformed fold changes) of select genes obtained from three independent experiments.

To determine whether the morphological alterations induced by CdS QDs were related to a transcriptional reprogramming of the gametogenesis process, a set of meiosis-specific genes expressed in different phases (early, middle, and late) of the sporulation process was analyzed by real-time PCR (Figure 7). Cells were grown for different time periods (3, 8, and 24 h) in the sporulation medium with (or without) the supplementation of CdS QDs, and total RNA was extracted from each sample; cDNAs were then synthesized and analyzed by real-time PCR using *ACT1* as the housekeeping gene.

Gene-expression analysis (Figure 7) showed a general down-regulation of regulatory genes (*IME2*, *NDT80* and *SMK1*) of the early–middle phase of the sporulation process and their downstream transcriptional targets (*SPO20* and *SPS100*). Ime2 is a positive regulator of Ndt80, the transcription factor necessary for the expression of *SMK1* and other middle–late genes required for the correct progression of meiosis and spore morphogenesis [25,26,27]. Therefore, it is possible that CdS QD treatment may interfere with the Ime2-dependent regulation of Ndt80, causing a blockage of the sporulation process.

**Figure 8:**
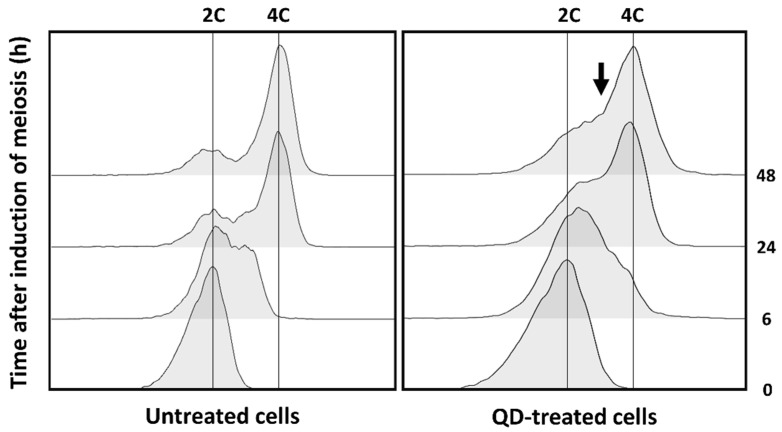
CdS QD treatment causes an alteration of the kinetic of meiotic-nuclear divisions during the sporulation process. DNA content determined by FC analysis in untreated cells and cells exposed to CdS QDs (5 mg L^−1^) for different amounts of time (0–48 h) in sporulation medium are shown. The *x*-axis represents DNA content (per cell), and the *y*-axis represents the numbers of cells in each condition. A high percentage of cells with an aberrant DNA amount (between 2C and 4C) was observed even at prolonged times of CdS QD exposure (indicated with black arrow).

To directly assess the effect of CdS QDs on meiotic cell-cycle progression, FC analysis of the DNA content was performed in cells grown for different amounts of time in the sporulation medium (Figure 8). Prior to induction of meiosis, yeast cells display the pattern typical of diploid cells with a DNA content of 2C (where C is the DNA content of a haploid genome; Figure 8). After 24 h of growth in the sporulation medium, most of the control (untreated) cells undergo DNA duplication, as indicated by the appearance of a peak corresponding to the tetraploid DNA content (4C; Figure 8). After CdS QD exposure in the sporulation medium, although the majority of cells present a DNA content of 4C (in line with the presence of multinucleated cells observed in these conditions), an unusually high percentage of cells with an aberrant DNA amount (between 2C and 4C) was observed even at prolonged times of QD exposure, indicating an altered kinetic of nuclear divisions in these samples (Figure 8). This finding suggests that the progression of DNA replication may also be affected by CdS QD treatment.

### 3.5. Identification of Hard Corona Proteins

To better understand how CdS QDs can inhibit the process of gametogenesis in yeast, we have also analyzed the composition of the hard protein corona of these NPs (Table 2), which is formed by yeast proteins that interact with high affinity to CdS QDs. Protein corona is known to affect the biological activity and environmental fate of ENMs [15,43]. Most of the hard corona proteins identified in the present work were involved in respiratory metabolism (Table 2), a metabolic process crucial for the sporulation process [44,45]. Other hard corona proteins identified were involved in glycolytic (Cdc19, Tdh3, Tdh2; Table 2) or translation processes (EF-1α; Table 2); these proteins were already identified in a previous work, performed with haploid yeast strains with the same NPs but in fermentative conditions [15]. Finally, among the proteins interacting with a high affinity to CdS QDs, we have identified Psa1 (Table 2), an essential protein required for polypeptide glycosylation and involved in cell-cycle progression, energy production, and the maintenance of cell wall integrity [46].

## 4. Discussion

Sporulation in *S. cerevisiae*, a crucial process to ensure viable recombinant progeny, is characterized by some tightly regulated key aspects conserved from yeast to higher organisms [21,22]. In this manuscript, we have observed that the treatment with CdS QDs caused an alteration in the meiotic-nuclear divisions, in combination with a strong inhibition of spore morphogenesis, with the formation of aberrant asci containing the meiotic products but without the formation of refractile spores (multinucleated cells). Notably, the formation of multinucleated giant cells, indicative of an altered spermatogenesis process, was also observed in rats and mice upon treatment with zinc-oxide- [17], silver- [19] and curcumin-loaded NPs [18]. In our work, we have observed that these morphological anomalies were not detected using other types of metal-based ENMs with different dimensions and zeta potentials (ZnS QDs, CuO NPs, and CeO_2_ NPs). However, we found that treatment with Cd(II) ions at much lower doses than those used for CdS QDs irreversibly blocks the meiotic-nuclear-division progression, without leading to the formation of multinucleated cells. It is known that Cd(II) has several harmful effects on the embryonic development in higher organisms [47,48,49]. Cd(II) can, in fact, be incorporated into the chromatin of developing gametes, which causes the failure of oocyte maturation in ovaries [48,49]. Exposure to high concentrations of Cd(II) can lead to the inhibition of the gametogenesis progression, with loss of cell adhesion and the induction of apoptosis [47].

**Figure 9:**
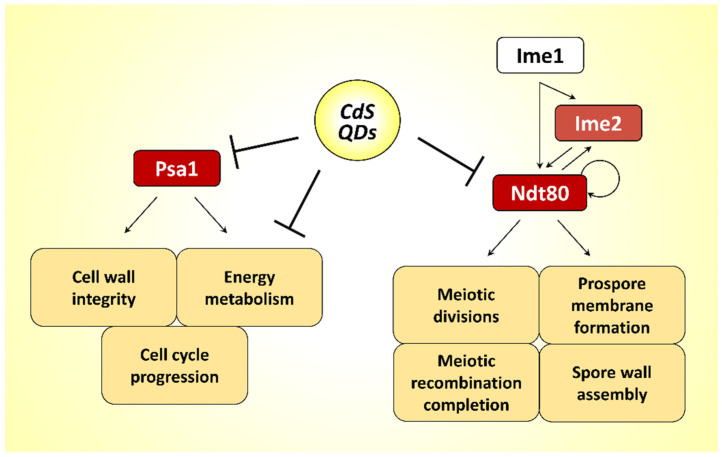
Schematic diagram illustrating the hypothetical mechanism of action of the CdS QDs in gametogenesis.

Notably, phenotypes similar to those observed after CdS QD treatment were also detected in some yeast strains, with mutations in sporulation-specific genes encoding for crucial components for the middle–late steps of gametogenesis process; in these mutants, the failure to form prospore membranes around the newly formed nuclei causes the formation of aberrant, multinucleated cells [24,30,31,32,33]. To determine whether the morphological alterations induced by CdS QDs were due to a transcriptional reprogramming of the gametogenesis process, a set of sporulation-specific genes was analyzed by real-time PCR. In agreement with the phenotypic effects observed in response to CdS QD exposure, down-regulation of crucial regulatory genes expressed in the early–middle phase of the sporulation process (*IME2*, *NDT80*, *SMK1*) and their downstream transcriptional targets was found. Among the genes most down-regulated, *NDT80* encodes a key regulator that coordinately controls the expression of the ∼200 genes required for exit from the meiotic prophase, progression from meiosis I to mesiosis II, and spore morphogenesis [50,51]. The induction of *NDT80* is tightly controlled by the transcription factor Ime1, the protein kinase Ime2, and Ndt80 itself, which triggers a positive feedback loop [23,50]. In addition, Ndt80 is required in turn for the induction of Ime2, promoting its activity to synchronize mitochondrial remodeling during meiosis [52]. In yeast, the expression at high levels of *NDT80* is essential to establish an irreversible commitment to nuclear division in the meiotic pathway [24]. It was, in fact, demonstrated that a reduced expression of this transcription factor (dependently by Ime2 and Ndt80 itself but not by Ime1) determines the loss of the meiotic commitment and a return to mitosis after meiosis I, without completing meiosis II, with the formation of aberrant, multinucleated cells [24]. A similar molecular mechanism can be hypothesized to also occur in yeast after treatment with CdS QDs (Figure 9). Future experiments will allow to better understand how CdS QDs can interfere with the expression of this transcriptional regulator, and if this effect is dependent on the action of Ime2. In agreement with these observations, flow-cytometry analysis showed that an altered kinetic of meiotic-nuclear divisions in sporulated cells exposed to CdS QDs.

Moreover, a reduced germination and a strongly altered morphology were observed in spores isolated from asci treated at concentrations (1 mg L^−1^) of CdS QDs that do not strongly compromise gametogenesis. It is interesting to note, among the proteins interacting with a high affinity to CdS QDs (and forming the protein corona of these NPs), we have identified Psa1, a protein required for the progression of the cell cycle and the formation of cell wall structure. Spores collected from a heterozygous yeast strain with a reduced dosage of *PSA1* gene showed decreased viability [46]. Considering that protein binding to ENMs is often associated with a reduction in the activity or misfolding of the identified adsorbed proteins [15,43], a similar outcome suggests that the interaction of CdS QDs with Psa1 could contribute to spore germination defects. Furthermore, the investigation of the hard corona protein composition showed that the most abundant proteins adsorbed with a high affinity on the QD surface were involved in energy metabolism, indicating that this molecular pathway can be impaired by the CdS QD-induced stress not only in fermentative conditions [12,13,15] but also in respiratory conditions. In yeast, exposure to CdS QDs affects the membrane potential and the morphology of the mitochondria and induces the production of reactive oxygen species (ROS) [12,13]. The respiratory metabolism is crucial for gametogenesis progression [44,45], and it is possible that an impaired energy metabolism can contribute to the deleterious effects on gametogenesis induced by CdS QD treatment (Figure 9).

## 5. Conclusions

In this manuscript, we have shown that CdS QDs alter the progression of meiosis and the spore formation in *S. cerevisiae*, and this effect is crucially related to the transcriptional repression of Ndt80 (Figure 9). It is interesting to note that Ndt80 is evolutionarily related to the metazoan family of Ig-fold transcription factors, including p53 which appears to have additional roles in spermatogenesis [53]. In nematodes, p53 seems, in fact, to be involved in the same molecular processes in which Ndt80 is involved in yeast, regulating the progression of meiosis, in particular chromosome segregation during meiosis I [53]. Thus, understanding the Ndt80-dependent molecular mechanism by which CdS QDs determine the loss of the meiotic commitment in yeast could be useful in preventing the toxicity of these ENMs in higher organisms. Thus, our manuscript shows how the yeast *S. cerevisiae* can be used as an ideal model organism, which allows a fast and reproducible analysis to identify the molecular mechanisms of toxicity of ENMs on the gametogenesis process.

## Figures and Tables

**Table 1:** List of primers used in this study.

Gene Name	Primer Sequence (5′–3′)	Amplicon Size (bp)
*ACT1*	FW: GAGGTTGCTGCTTTGGTTATTGA RE: CGTCGTCACCGGCAAAA	67
*IME1*	FW: CGTTGAAAAATCACCACCGCCA RE: CTGAAGGAGTAAGCCGCAGCA	111
*IME2*	FW: ACGGCCTACGTTTCCACAAGAT RE: CCACGCACCCGAATGCCCAA	100
*NDT80*	FW: GCCATCAATGGCGCAGCCGT RE: CGAGATGGAGGCCCCAGAGT	106
*SMK1*	FW: TGACCAGCTCGCCCTATGACG RE: CCGAGAGCTGCACGGACGAAT	93
*SPO1*	FW: TGGATTATCAGGCGGAAGTTGG RE: TCCTCTTCAAGGTCCCACTCTT	93
*RIM4*	FW: GGCAAAACATTTACAGGGCCAG RE: GCTTTCCTGCTGGGATCCGC	90
*DIT1*	FW: GGTCGATGATGACGTCGTGAG RE: AGCCAATGGCGTCAACACCAG	89
*DIT2*	FW: CGTGCAAGTTGGGGGCGGAA RE: GCCCCAAGTTTTGGGATCGTG	92
*SPO20*	FW: TCACCCAAACTGTCGGTTCGATGA RE: GTAGCAAGGCCATCCCTTTCG	90
*SPS100*	FW: ACGCGGAAGGTAGAGGCACTT RE: CCTGTGGGCGTTTTGTCTGGT	100

**Table 2:** Protein composition of the hard protein corona of CdS QDs.

Protein	Description (UniProt Accession N.)	Score ^1^	Coverage ^2^	Molecular Weight (KDa)	pI	Biological Process
Atp1	Alpha subunit of the mitochondrial ATP synthase (P07251)	132.45	52%	59	9.5	Energy metabolism
Cdc19	Pyruvate kinase (P00549)	192.37	83%	55	7.8	Energy metabolism
Idp2	Isocitrate dehydrogenase (P41939)	174.19	77%	47	6.1	Energy metabolism
Lat1	Dihydrolipoamide acetyltransferase component of the pyruvate dehydrogenase complex (P12695)	79.57	38%	52	8.0	Energy metabolism
Pdb1	E1 beta subunit of the pyruvate dehydrogenase complex (P32473)	73.89	36%	40	5.0	Energy metabolism
Tdh2	Glyceraldehyde-3-phosphate dehydrogenase (P00358)	137.4	55%	36	7.0	Energy metabolism
Tdh3	Glyceraldehyde-3-phosphate dehydrogenase (P00359)	149.31	72%	36	7.0	Energy metabolism
Leu2	Beta-isopropylmalate dehydrogenase (P04173)	81.46	58%	39	5.5	Metabolic process
Psa1	Mannose-1-phosphate guanyltransferase (P41940)	73.16	37%	40	6.3	Metabolic process
EF-1α (Tef1/2)	Translational elongation factor EF-1 alpha (P02994)	93.01	44%	50	9.1	Translation

^1^ The probability that the observed match is not a random event. ^2^ The ratio (%) between the number of amino-acid residues covered in the identified peptides and the total number of residues in the protein sequence.

## Data Availability

Not applicable.

## Supplementary Materials

The following supporting information can be downloaded at: https://www.mdpi.com/article/10.3390/nano12132208/s1. Supplementary Methods, Figure S1: Characterization of ENMs used in the present work. Supplementary Methods, Figure S2. Powder X-ray diffraction pattern of CdS QDs. References [28,29,39,42] have been cited in the Supplementary Materials.
