# Supplementary material for: Cadmium Sulfide Quantum Dots Adversely Affect Gametogenesis in Saccharomyces cerevisiae
Source: Nanomaterials (Basel). 2022 Jun 27;12(13):2208. doi: 10.3390/nano12132208 (PMC9268033; doi:10.3390/nano12132208)
Supplement: Supplementary file 1 [file nanomaterials-12-02208-s001.zip › nanomaterials-1755867-supplementary.pdf]

Supplementary material

Cadmium Sulfide Quantum Dots Adversely Affect Gametogenesis in *Saccharomyces cerevisiae*

Riccardo Rossi [1,†], Roberta Ruotolo [1,*,†], Giuseppe De Giorgio [1], Marta Marmiroli [1], Marco Villani [2], Andrea Zappettini [2] and Nelson Marmiroli [1,3,*]

[1] Department of Chemistry, Life Sciences and Environmental Sustainability, University of Parma, Parco Area delle Scienze 11/A, 43124 Parma, PR, Italy; riccardo.rossi1@unipr.it (R.R.); giuseppe.degiorgio@unipr.it (G.D.G.); marta.marmiroli@unipr.it (M.M.)

[2] Institute of Materials for Electronics and Magnetism (IMEM), National Research Council (CNR), Parco Area delle Scienze 37/A, 43124 Parma ,PR, Italy; marco.villani@imem.cnr.it (M.V.); andrea.zappettini@imem.cnr.it (A.Z.)

[3] The Italian National Interuniversity Consortium for Environmental Sciences (CINSA), Parco Area delle Scienze 93/A, 43124 Parma, PR, Italy

* Correspondence: roberta.ruotolo@unipr.it (R.R.); nelson.marmiroli@unipr.it (N.M.); Tel.: +39-0521-906283 (R.R.); +39-0521-905606 (N.M.)

† These authors contributed equally to this work.

Supplementary Methods

Synthesis and characterization of CdS QDs

Cadmium acetate (99%), N,N-dimethyl formamide (99%) and thiourea (99.5%) were purchased from Sigma-Aldrich (Merck, Darmstadt, Germany). CdS QDs were synthesized by IMEM-CNR as previously described [1]. Briefly, 10 ml of 10^5 M thiourea solution were added to 100 ml dimethylformamide (DMF). The solution was then heated at 70 °C, 10 ml of 10^5 M solution of cadmium acetate were added and the temperature raised to 90 °C. The reaction was stopped after 5 minutes, the solid phase was washed with water and collected by centrifugation.

Transmission electron microscopy (TEM) analysis (Figure S1a) was performed using a Talos F200S G2 SEM FEG (Thermo Fisher Scientific, Waltham, MA, USA). Hydrodynamic diameter (nm) and zeta potentials (mV) were determined by dynamic light scattering (DLS) analysis at room temperature (Figure S1b) using a Zetasizer Nano ZSP (Malvern Instruments, Malvern, UK). The crystal structure of CdS QDs was examined with powder X-ray diffraction (XRD) spectrum (Figure S2) performed using ARL™ X'Tra diffractometer (Thermo Fisher Scientific, Wyman Street, Waltham, MA, USA; Cu Kα source, Bragg-Brentano geometry).

Synthesis of ZnS QDs

The synthesis of ZnS QDs was adapted from [2]. Briefly, 10 mmol of anhydrous $ZnCl_2$ was dissolved into 200 mL of ethylene glycol and heated to 160 °C under Ar atmosphere (solution #1). In another flask, purged with Ar, 10 mmol of thiourea was dissolved into 50 mL of ethylene glycol at 80 °C (solution #2). Under vigorous magnetic stirring, solution #2 was then quickly added into solution #1 with the formation of ZnS nanocrystals. This solution was allowed to rest at 150 °C for 10 min and then brought to room temperature using an ice bath. The ZnS nanocrystals were separated from the reaction solution by centrifugation, washed with acetone and ethanol, and finally dried in a desiccator.

Supplementary Figures

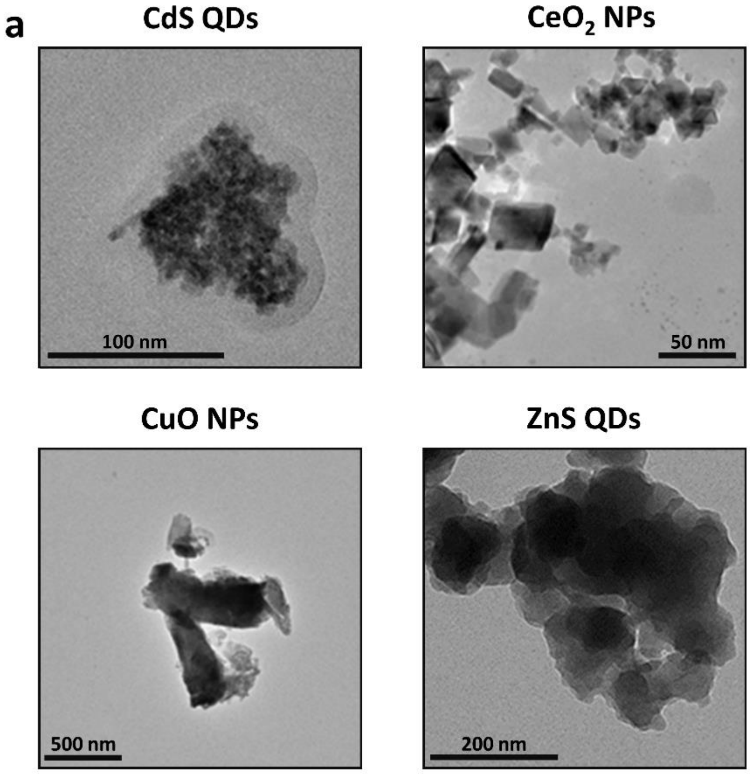

b

ENMs	Size (nm)	Zeta potential (mV)	Hydrodinamic diameter (nm)
CdS QDs	<5	-22	196.5
CeO_2 NPs	<40	40	246.9
CuO NPs	<25	-25	533.9
ZnS QDs	<5	12	302.9

Figure S1. Characterization of ENMs used in the present work. (**a**) Representative TEM images of the CdS QDs, CeO_2 NPs, CuO NPs and ZnS QDs (images adapted from [3] and [4]). (**b**) Physical characterization of the ENMs used in the present work.

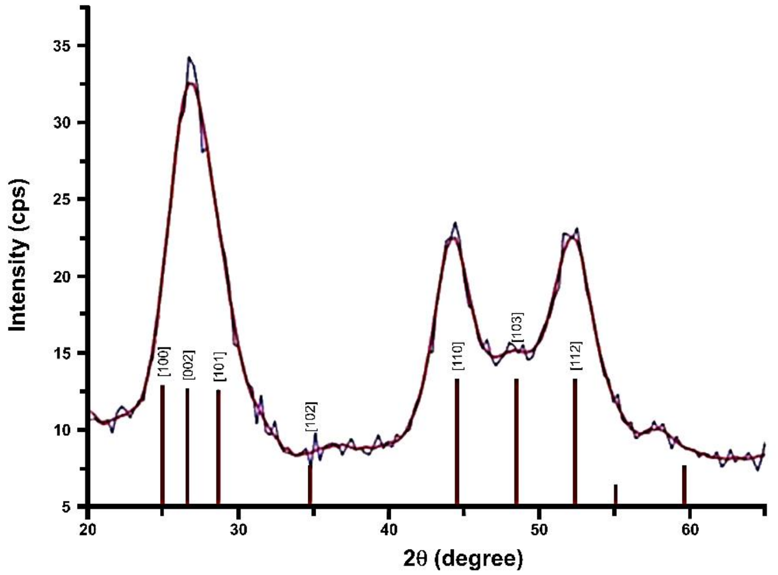

Figure S2. Powder X-ray diffraction pattern of CdS QDs. The line spectra show the hexagonal crystal structure of the CdS QDs.

Bibliography

1. Villani, M.; Calestani, D.; Lazzarini, L.; Zanotti, L.; Mosca, R.; Zappettini, A. Extended functionality of ZnO nanotetrapods by solution-based coupling with CdS nanoparticles. *J. Mater. Chem.* **2012**, *22*, 5694–5699.
2. Zhao, Y.; Zhang, Y.; Zhu, H.; Hadjipanayis, G.C.; Xiao, J.Q. Low-temperature synthesis of hexagonal (Wurtzite) ZnS nanocrystals. *J. Am. Chem. Soc.* **2004**, *126*, 6874–6875.
3. Pagano, L.; Marmiroli, M.; Villani, M.; Magnani, J.; Rossi, R.; Zappettini, A.; White, J.C.; Marmiroli, N. Engineered Nanomaterial Exposure Affects Organelle Genetic Material Replication in Arabidopsis thaliana. *ACS nano* **2022**, *16*, 2249–2260.
4. Marmiroli, M.; Pagano, L.; Rossi, R.; De La Torre-Roche, R.; Lepore, G.O.; Ruotolo, R.; Gariani, G.; Bonanni, V.; Pollastri, S.; Puri, A. et al. Copper Oxide Nanomaterial Fate in Plant Tissue: Nanoscale Impacts on Reproductive Tissues. *Environ. Sci. Technol.* **2021**, *55*, 10769–10783.
